# Potential of *Bacillus subtilis* PB6 in corn-based diets to combat subclinical necrotic enteritis in broilers

**DOI:** 10.1016/j.psj.2025.105574

**Published:** 2025-07-13

**Authors:** Most Khairunnesa, Alip Kumar, Kosar Gharib-Naseri, Mingan Choct, Reza Barekatain, Shu-Biao Wu

**Affiliations:** a School of Environmental and Rural Science, University of New England, Armidale, NSW 2351, Australia; b SARDI Roseworthy campus, University of Adelaide, SA 5371, Australia

**Keywords:** *Bacillus subtilis*, Growth performance, Histomorphology, Necrotic enteritis, Broiler

## Abstract

*Bacillus*-based probiotics and dietary enzymes have gained attention for supporting gut health, nutrient digestion, and intestinal integrity in poultry. This study evaluated the effects of *Bacillus subtilis* PB6 (**Pb**) and xylanase (**Xy**), individually and in combination, on the performance, intestinal lesions, and histomorphology of broiler chickens challenged with subclinical necrotic enteritis (**SNE**). A total of 630-d-old, mixed-sex Cobb 500 broilers were utilized in a 2 × 2 + 1 factorial arrangement of treatments: NE challenge without additives (**CC**); NE challenge with Xy at 0.03 % (Xy); NE challenge with Pb at 0.05 % (Pb); NE challenge with Xy at 0.03 % and Pb at 0.05 % (Xy+Pb); and, a non-challenge, additive free control (**NC**). In the pre-challenge period, birds in NC and CC groups were considered a control. A significant Xy × Pb interaction was observed for body weight gain (**BWG**) (*P* < 0.01) and FCR (*P* < 0.05) before challenge (d0-8). Pb efficacy for reducing FCR was significant without Xy supplementation, while Xy enhanced BWG without Pb (*P* < 0.01). During d9-19, all challenged birds showed reduced BWG (*P* < 0.05) and increased FCR (*P* < 0.05) compared to NC birds. During d20-35 and d0-35, Pb significantly improved FCR and reduced feed intake (**FI**) as the main effect among challenged birds (*P* < 0.05), equating the performance of NC birds (*P* > 0.05). NE challenge increased (*P* < 0.05) duodenal lesions in females but not males. A significant Xy × Pb interaction (*P* < 0.05) was observed for villus height (**VH**) and the VH to crypt depth (**CD**) ratio. Pb enhanced VH and VH/CD (*P* < 0.05) only when Xy was not supplemented. Furthermore, Pb-fed birds showed a trend towards increased villus surface area (**VSA**) (*P* = 0.067). In conclusion, Pb improves feed efficiency and mitigates the adverse effects of SNE-induced damage to intestinal health, while Xy enhances pre-challenge growth without augmenting Pb’s benefits during NE.

## Introduction

The global restriction on antibiotic use in animal feed has led to the re-emergence of diseases such as necrotic enteritis (**NE)**, which was previously well-managed through antibiotic supplementation in the feed. Antibiotics not only enhanced growth performance but also maintained gut health by modulating the microbiota, suppressing harmful bacteria, reducing fermentation, and limiting intestinal inflammation (Carlson and Fangman, 2018; Gaskins et al., 2002). Their withdrawal disrupted these protective mechanisms, allowing opportunistic pathogens like *Clostridium perfringens* to proliferate, especially in intensive poultry systems. This disease results in significant production loss because of its detrimental impact on growth performance and feed efficiency, with estimated annual economic losses of around US $6 billion globally (Wade and Keyburn, 2015). Although both the clinical and subclinical forms of NE pose a serious threat to poultry profitability, subclinical NE (**SNE**), characterized by mild intestinal lesions and low mortality, can be particularly devastating as its symptoms often go unnoticed, but result in impaired feed efficiency and reduced weight gain (Gharib-Naseri et al., 2019; Lovland and Kaldhusdal, 2001; M'Sadeq et al., 2015; Timbermont et al., 2011). Antibiotics have traditionally been used in intensive poultry production to enhance growth performance and improve intestinal health (Castanon, 2007). However, their use in animal production has led to the development of antibiotic-resistant bacteria, which pose a risk of transmission between animals and humans (He et al., 2020; Liu et al., 2021b; Martin et al., 2015; Yang et al., 2022; Zhang et al., 2015). As many countries have banned antibiotic use in poultry feed, there is an urgent need for alternative strategies to maintain broiler performance and gut health.

Among the promising alternatives, *Bacillus-*based probiotics have gained significant attention due to their ability to modulate gut microbiota, enhance nutrient digestibility, and improve intestinal integrity (Naeem and Bourassa, 2025). Among them, *B. subtilis* has shown substantial probiotic potential. *Bacillus* species, known for their rapid colonization and proliferation in the chickens' gastrointestinal tract, exert beneficial effects through the competitive exclusion of pathogens and production of antimicrobial substances (Cartman et al., 2008; Latorre et al., 2014). Dietary supplementation of this probiotic has been associated with many positive outcomes, including growth performance and enhanced intestinal morphology (Qiu et al., 2021; Samanya and Yamauchi, 2002), increased serum immunoglobulin levels, and improved disease resistance, particularly against *Escherichia coli* (Guo et al., 2020). Further evidence shows *B. subtilis* can stimulate anti-inflammatory cellular responses, strengthen intestinal barrier function, and modulate microbial composition and fermentative activity in a beneficial manner (Vieco-Saiz et al., 2024). A key factor behind these benefits is that this group of bacteria produces a range of enzymes, including α-amylase (Ibrahim et al., 2012), β-glucanase (Aono et al., 1992), xylanase (Monisha et al., 2009), protease (Olajuyigbe and Ajele, 2005), lipase (Shah, 2011), phytase (Choi et al., 2001), and cellulase (Hendricks et al., 1995), which may help to degrade complex antinutritional factors in poultry diets and improve nutrient absorption (Latorre et al., 2015). Beyond poultry, *Bacillus*-based probiotics have shown positive effects in aquaculture, where they have been reported to enhance growth, immune responses, and the activity of key antioxidant enzymes, including catalase, superoxide dismutase, and glutathione peroxidase in fish (Naveed et al., 2024). Furthermore, *B. subtilis* spores are highly resilient to digestive conditions (Teo and Tan, 2003), making them ideal candidates for feed additive applications. This bacterium also shows good stability in high temperatures during manufacturing, processing, and storage compared to other probiotic species (Ramlucken et al., 2020). In addition to their enzyme-producing capabilities and resilience, *B. subtilis* PB6 has demonstrated beneficial effects in improving growth performance, enhancing intestinal morphology, and helping control diseases such as NE in broilers. However, studies have significant inconsistencies regarding the parameters evaluated and their results. For example, Aljumaah et al. (2020) reported an improvement in the feed conversion ratio (**FCR**) during d28–35, but they observed no significant changes in ileal histomorphometry measurements. In contrast, Liu et al. (2021a) found no improvement in FCR under similar conditions. On the other hand, Jayaraman et al. (2013) observed an improvement in FCR at d35, along with reduced intestinal lesions and improved duodenal villus height (**VH**) and villus height/crypt depth (**VH/CD**) ratio. Hassan et al. (2018) also observed an overall improvement in FCR; however, their study did not include the NE challenge. These inconsistencies highlight the need for further research to better understand and clarify the effects of *B. subtilis* PB6, particularly under NE challenged conditions.

Dietary inclusion of exogenous enzymes, particularly xylanase, has become widely recognised for enhancing feed efficiency and improving broiler performance. Xylanase targets non-starch polysaccharides (**NSP**), in particular, arabinoxylans in plant-based feedstuffs used for pig and poultry nutrition. By degrading arabinoxylans, xylanase improves nutrient utilization (Kim et al., 2022) and promotes gut health through the production of prebiotic xylooligosaccharides (Dhaver et al., 2023; Immerzeel et al., 2014; Thakur et al., 2022). These prebiotics promote gut health by inhibiting harmful pathogens, such as *C. perfringens,* and promoting the growth of beneficial bacteria, such as *Lactobacillus* spp. and *Bifidobacteria* spp. (Lin et al., 2016; Nieto-Domínguez et al., 2017; Petry et al., 2021). In several studies, xylanase supplementation has been shown to positively affect performance, nutrient bioavailability, and FCR, as well as reduce the number of *C. perfringens* in the small intestine of chickens fed wheat-based diets (Choct et al., 2006; Gao et al., 2008; Jackson et al., 2003). Recently, Daneshmand et al. (2023a) reported the beneficial effects of xylanase on broiler performance during the finisher phase under an *Eimeria* spp. challenge in a wheat-based diet. Furthermore, Van Hoeck et al. (2021) highlighted the positive effects of xylanase on performance and carcass traits in broilers fed wheat-based diets without any disease challenge. While the benefits of xylanase in wheat-based diets are well documented, its effects on corn-soybean-based diets, particularly under NE challenge conditions, are not as extensively studied.

Recent studies have highlighted the potential additive effects of combining *Bacillus*-based probiotics and dietary xylanase. For example, Sultan et al. (2024) observed improved growth, nutrient digestibility, immune response, and caecal microbiota in Japanese quail supplemented with both additives. Similarly, Nusairat et al. (2018) reported improvements in FCR and reduced naturally occurring NE-caused intestinal lesion scores in broilers receiving a diet supplemented with a combination of *Bacillus*-based probiotics and xylanase. These findings suggest that the concurrent use of probiotics and xylanase may support host gut health by fostering beneficial microbial populations and establishing a balanced gut microbiota. In light of these findings, the current study aimed to investigate the potential of xylanase and *B. subtilis* PB6 on growth performance, liveability, gut histology, and intestinal lesions in broilers with SNE challenge. This study was based on two hypotheses: 1) the addition of xylanase or *B. subtilis* PB6 individually mitigates the adverse effects of SNE by enhancing feed efficiency and improving gut health, and 2) the combination of xylanase and *B. subtilis* PB6 exerts a synergistic effect, alleviating the negative impacts of SNE, and resulting in higher productivity compared to their individual use.

## Materials and methods

### Animal ethics

The experimental procedures applied in the current study were approved by the Animal Ethics Committee of the University of New England, Armidale, NSW 2351, Australia (Approval number ARA22-005). The experiment was conducted following the guidelines for the care and use of laboratory animals for scientific purposes, accredited by the Australian Bureau of Animal Health (NHMRC, 2013).

### Birds and husbandry

A total of 630 d-old mixed-sex Cobb 500 broiler chicks were obtained from Baiada hatchery in Tamworth, NSW, Australia. After arrival, birds were weighed and assigned randomly to 45-floor pens, ensuring no significant difference in initial pen weight over the experimental treatments. Birds were housed in an environmentally controlled facility with a littered floor, using softwood shavings as bedding (approximately 8 cm deep). One tube feeder and three nipple drinkers were used in each pen to allow *ad libitum* feeding and drinking. Temperature, humidity, and lighting schedules were maintained according to Cobb 500 management guidelines (Cobb500, 2022b).

### Feed additives

This study evaluated the potential of two feed additives supplied by Kemin Animal Nutrition and Health, Singapore, to improve the performance and intestinal health of broilers challenged with SNE. The feed additives used were xylanase and *B. subtilis* PB6. This xylanase is a thermostable, monocomponent enzyme produced by *Thermopolyspora flexuosa,* expressed in *Pichia pastoris*. It is a beta-1,4 endo-xylanase enzyme belonging to the GH11. It has a minimum enzyme activity of 3,000,000 U/g formulated on a corn starch-based carrier, with a recommended dose of 10g/t, corresponding to an activity of 30,000 U/kg. *B. subtilis* PB6, a unique strain of *B. subtilis*, is a naturally occurring spore-forming microorganism that is stable during feed processing. It is included at an inclusion rate of 2.2 × 10^8^ CFU/g or 1 × 10^8^ /kg of feed.

### Experimental design and dietary treatments

The experimental birds were randomly assigned to a 2 × 2 + 1 arrangement of treatments, including 5 treatment groups with nine replicates per treatment and 14 birds per pen. The treatments applied in this study are shown in Table 1. Briefly, the treatments were: 1) NE challenge without additives (**CC**); 2) NE challenge with Xy at 0.03 % (**Xy**); 3) NE challenge with Pb at 0.05 % (**Pb**); 4) NE challenge with Xy at 0.03 % and Pb at 0.05 % (Xy+Pb); and, 5) non-challenge with no additives (**NC**). Before the challenge (d0-8), NC and CC were collectively considered as a control group. All diets were corn-soybean-based and supplemented with phytase, where matrix values of phytase were considered at 500 FTU/kg (100 g/t). The nutrient composition of the feed ingredients was analyzed before diet formulation by near-infrared spectroscopy (AminoNIR®, Evonik Amino Prox, Essen, Germany). The diets were formulated according to the Cobb 500 nutritional recommendations for broilers (Cobb 500, 2022a). Feed was provided as crumbles during the starter phase (d0-8) and pellets during the grower (d9-20) and finisher (d20-35) phases. The details of the diet composition for each phase and nutrient contents are shown in Table 2.

**Table 1:** Treatments applied in this study.

1Treatments	Feed additives		Inclusion level, %	2Necrotic enteritis challenge
	Xylanse (Xy)	*Bacillus subtilis* PB6 (Pb)		
CC	No	No	-	Challenged
Pb	No	Yes	0.05	Challenged
Xy	Yes	No	0.03	Challenged
Xy+Pb	Yes	Yes	0.03+0.05	Challenged
NC	No	No	-	Non-challenged

1 CC: challenged control, Xy: challenged control+ xylanase (0.03 %), Pb: challenged control+ *B. subtilis* PB6 (0.05 %), Xy + Pb: challenged control+ xylanase (0.03 %) +*B. subtilis* PB6 (0.05 %), NC: non-challenged control.

2 NE-challenged birds were orally gavaged with *Eimeria* spp. on d 9 and *Clostridium perfringens* on d 14 and 15.

**Table 2:** Diet composition (as-fed basis, %), and calculated nutrients.

Ingredients (%)	Starter (d0-8)	Grower (d9-19)	Finisher (d20-35)
Corn	58.1	62.5	67.2
Soybean meal	36.6	31.6	26.9
Canola oil	1.55	1.80	2.10
Dicalcium Phosphate	1.44	0.97	0.90
Limestone	0.96	0.75	0.64
DL-methionine	0.34	0.32	0.30
Salt	0.26	0.26	0.24
L-lysine HCl 78.4	0.21	0.24	0.28
L-threonine	0.15	0.11	0.12
Choline Cl 60 %	0.09	0.12	0.14
1Mineral Premix	0.08	0.08	0.08
2Vitamin Premix	0.08	0.08	0.08
Sodium bicarbonate	0.03	0.03	0.03
3Phytase	0.01	0.01	0.01
4Sand	0.08	1.17	0.98
5**Calculated nutrients (%, otherwise as indicated)**			
Dry Matter	89.0	89.0	88.8
AME (kcal/kg)	2,950	3,000	3,075
Crude protein	22.7	20.7	18.9
Crude fat	3.90	4.22	4.61
Crude fiber	3.24	3.12	3.03
Digestible Arg	1.34	1.20	1.07
Digestible Lys	1.26	1.16	1.08
Digestible Met	0.61	0.57	0.54
Digestible Meth+Cyst	0.94	0.88	0.83
Digestible Trp	0.28	0.26	0.23
Digestible Iso	0.87	0.78	0.70
Digestible Thr	0.90	0.79	0.74
Digestible Val	0.93	0.84	0.77
Calcium	0.96	0.80	0.74
Available phosphorus	0.54	0.40	0.37
Sodium	0.19	0.18	0.17
Potassium	1.10	1.01	0.93
Chloride	0.25	0.26	0.26
Choline mg/kg	1,716	1,700	1,704
Linoleic 18:2	1.50	1.59	1.70

AME=apparent metabolizable energy.

1 Trace mineral concentrate supplied per kilogram of diet: Cu (sulfate), 16 mg; Fe (sulfate), 40 mg; I (iodide), 1.25 mg; Se (selenate), 0.3 mg; Mn (sulfate and oxide), 120 mg; Zn (sulfate and oxide), 100 mg; cereal-based carrier, 128 mg; mineral oil, 3.75 mg.

2 Vitamin premix per kg diet:vitamin A, 12 MIU; vitamin D, 5 MIU; vitamin E, 75 mg; vitamin K, 3 mg; nicotinic acid, 55 mg; pantothenic acid, 13 mg; folic acid, 2 mg; riboflavin, 8 mg; cyanocobalamin, 0.016 mg; biotin, 0.25 mg; pyridoxine, 5 mg; thiamine, 3 mg; antioxidant, 50 mg.

3 Phytase: Quantum Blue, 5GAB Vista, 500 FTU/kg diet.

4 Sand was replaced with the required amount of xylanase and *B. subtilis* PB6 and added to the top.

5 Nutrient contents were measured using near-infrared spectroscopy (NIRS, Evonik Amino Prox, Germany).

### Birds sexing

On d5, the feathers of a subset of 6 birds from each pen were collected for sex determination using feather DNA sexing with high-resolution melting curve (**HRM**) analysis (England et al., 2021). This ensures that at least one male and one female from each pen will be sampled.

### NE challenge

The current investigation followed the previous NE model (Rodgers et al., 2015, Wu et al., 2014), in which *Eimeria* spp. vaccine strains and *C. perfringens* were used to induce SNE. Briefly, challenged birds were gavaged with 1 mL *Eimeria spp*. including 5000 *Eimeria acervulina* and *E. maxima* oocysts and 2500 *E. brunetti* oocysts (Eimeria Pty Ltd., Ringwood, VIC, Australia) orally. On d14 and 15, challenged birds were orally gavaged with 1 mL *C. perfringens* (EHE-NE18) containing approximately 10^8^ CFU (CSIRO Livestock, Geelong, VIC, Australia). Non-challenged birds received a 1 mL oral gavage of phosphate-buffered saline (**PBS**) on d9 and 1 mL sterile thioglycolate broth on d14 and 15, respectively, as a sham challenge. Any deaths following the NE challenge were followed by necropsy to determine the cause of death. Throughout the experiment, all dead birds were recorded for sex and weight.

### Performance measurements and sampling

Body weight (**BW**) and feed intake (**FI**) were recorded on d8, d19, d28, and d35, and BWG, FI, and FCR were calculated accordingly. Daily mortality and body weight of dead birds were recorded, and the FCR was adjusted according to the mortality. FI was determined based on the dry matter (**DM**) content of the feed (both feed-in and feed-out). The FCR was also calculated on a DM basis. However, both feed intake and FCR values are presented on an 88 % DM basis (Noblet et al., 2015). On d35, all remaining birds were dissected to identify their sex by visual inspection of testes, and the number of male and female birds in each pen was recorded. On d16, 2 birds (one male and one female) from each pen were randomly selected, weighed, stunned by an electric stunner (JF poultry equipment, Weltevreden Park, South Africa), and decapitated for sampling.

### Intestinal lesion scoring

Sampled birds were scored for NE lesions in the duodenum, and jejunum by visual examination following a previously reported lesion scoring system (Keyburn et al., 2006; Shojadoost et al., 2012) with a lesion score range from 0 to 6, where 0 illustrated no lesions, and 6 emerged as the most severe and acute intestinal lesions.

### Histomorphology

On d16, a 5 cm segment of jejunal tissue from one bird in each pen was excised, flushed with PBS, and preserved in 10 % neutral buffered formalin (**NBF**) until further use. Tissue sections (5 µm) were processed using standard Haematoxylin and Eosin staining described by Golder et al. (2011) for histomorphology analysis. Slides were scanned (Hamamatsu Nano Zoomer 2.0 RS Slide Scanner, Japan), and parameters were measured using NDP. View. 2.5 software (Hamamatsu Photonics K.K., Higashi-ku, Hamamatsu, Japan). Histological parameters, namely, VH, CD, and villus width (**VW**), were measured from 10 randomly selected intact villi and associated crypts on one section per chicken. Apparent villus surface area (**VSA**) was calculated using the previously described formula VSA = 2π (VW/2) (VH) by Sakamoto et al. (2000).

### Statistical analysis

All data collected in this study were first tested for normal distribution and analyzed in JMP 16.0 (SAS Institute, Cary, NC, USA). Normally distributed performance data during d0-8 were analyzed in a completely randomized design using a 2 × 2 factorial arrangement. Those involved challenges at the later stages were analyzed using a 2 × 2 factorial arrangement followed by a Student’s t-test to compare main effects. When interactions were observed, one-way ANOVA was performed, and paired comparison was conducted using Tukey’s test, which also involved NC. The female percentage (corrected to dead birds) was used as a covariate in the performance data analysis when a significant sex effect was observed. Intestinal lesion scoring data were analyzed using the non-parametric Kruskal Wallis (Wilcoxon test) test as the data were not normally distributed. Differences between means were considered statistically significant when the *P*-value was < 0.05 and were considered to show a tendency towards significance with 0.05 < *P* < 0.1.

## Results

### Growth performance and liveability

Table 3 shows the effect of xylanase and *B. subtilis* supplementation on the growth performance before challenge (d0-8). During the initial growth period (d0-8), before the NE challenge, the supplementation of Xy and Pb showed a significant interaction on BWG and FCR. It was shown that the extent of Pb in reducing the FCR was higher without than with Xy supplementation. Xy increased BWG (*P* < 0.01) without Pb but did not exhibit any effect with Pb supplementation. Moreover, the presence of Pb in the diet reduced (*P* < 0.001) FI. Table 4, Table 5 show the performance results during and after the NE challenge. Post-NE challenge, no interaction between Xy and Pb was observed regarding performance. From d9-19, all NE-challenged birds exhibited reduced BWG and higher FCR (*P* < 0.05) compared to the NC birds. Combining the starter and grower phases on d0-19, the FCR of birds supplemented with Pb shifted towards NC birds from CC, showing no difference (*P* > 0.05) either from NC and CC. During the challenge recovery phase on d20-28, an interaction between Xy and Pb tended to be significant (*P* = 0.085), with Pb reducing FI only when Xy was not supplemented. Moreover, in this phase, the supplementation of Pb as a main effect reduced FI (*P* < 0.05) and FCR (*P* < 0.001). No treatment effects or Xy × Pb interactions were observed during d29-35 (*P* > 0.05). Throughout the finisher phase (d20–35) and over the entire experimental period (d0-35), Pb supplementation reduced FI (*P* < 0.05) and FCR (*P* < 0.01) as a main effect, and no interactions were shown (*P* > 0.05). Over the entire course of the study (d0-35), CC birds showed lower BWG and higher FCR (*P* < 0.05) compared to the NC birds. However, the FCR of birds receiving Pb did not significantly differ from NC birds during either the finisher (d20–35) and overall study (d0-35) period (*P* > 0.05). Liveability remained unaffected (*P* > 0.05) across all treatments and study phases (data not shown).

**Table 3:** Effect of xylanase and *B. subtilis* PB6 on the growth performance of broilers before challenge (d0-8) with necrotic enteritis.

1Treatments	Xylanase (%)	*B. subtilis* (%)	(d0-8)		
			FI (g)	BWG (g)	FCR (g/g)
Control	No	No	248	187^b^	1.325^a^
Pb	No	Yes	219	189^b^	1.164^b^
Xy	Yes	No	253	197^a^	1.290^a^
Xy+Pb	Yes	Yes	220	185^b^	1.192^b^
2SEM			2.43	1.75	0.013
**Main effect (2×2)**					
Xylanase (Xy) %					
0			234	188	1.244
0.03			238	191	1.241
SEM			1.73	1.25	0.011
*B. subtilis* (Pb) **%**					
0			251^a^	192^a^	1.307^a^
0.05			220^b^	187^b^	1.177^b^
SEM			1.73	1.25	0.011
***P*-value**					
Xy			0.171	0.106	0.759
Pb			<0.0001	0.006	<0.0001
Xy × Pb			0.445	0.001	0.015

FI: feed intake, BWG: body weight gain, FCR: feed conversion ratio.

FI and FCR values were standardized for 88 % dry matter.

^a-b^values within a column with no common superscripts differ significantly (*P* < 0.05).

1 Control: no additives, Xy: xylanase (0.03 %), Pb: *B. subtilis* PB6 (0.05 %), Xy + Pb: xylanase (0.03 %) +*B. subtilis* PB6 (0.05 %).

2 SEM: standard error of mean.

**Table 4:** Effect of xylanase and *B. subtilis* PB6 on the growth performance of broilers challenged with necrotic enteritis at different growth stages (d9-19, d0-19, and d20-28).

1Treatments	2NE	Xylanase (%)	*B. subtilis* (%)	(d9-19)			(d0-19)			(d20-28)		
	Challenge			FI (g)	BWG (g)	FCR (g/g)	FI (g)	BWG (g)	FCR (g/g)	FI (g)	BWG (g)	FCR (g/g)
CC	Yes	No	No	942	654^b^	1.443^a^	1188^ab^	841^b^	1.413^a^	1434^a^	886^ab^	1.619^a^
Pb	Yes	No	Yes	923	635^b^	1.454^a^	1140^b^	824^b^	1.384^ab^	1336^b^	870^b^	1.538^bc^
Xy	Yes	Yes	No	947	648^b^	1.463^a^	1198^a^	844^b^	1.419^a^	1370^ab^	860^b^	1.589^ab^
Xy+Pb	Yes	Yes	Yes	914	631^b^	1.450^a^	1132^b^	816^b^	1.387^a^	1367^ab^	903^ab^	1.518^c^
NC	No			964	726^a^	1.328^b^	1213^a^	913^a^	1.329^b^	1427^a^	933^a^	1.533^bc^
^3^SEM				12.8	8.28	0.017	14.1	9.38	0.014	20.7	15.3	0.015
***P*-value**				0.062	<0.0001	<0.0001	<0.001	<0.0001	0.001	0.005	0.018	<0.0001
**Main effect (2×2)**												
Xylanase (Xy) %												
0				933	644	1.448	1164	833	1.399	1385	878	1.578
0.03				931	639	1.457	1165	830	1.403	1372	884	1.553
SEM				9.48	5.82	0.011	10.5	6.51	0.011	14.1	11.0	0.010
*B. subtilis* (Pb) **%**												
0				944	651^a^	1.453	1193^a^	843^a^	1.416	1415^a^	883	1.604^a^
0.05				918	633^b^	1.452	1136^b^	820^b^	1.386	1343^b^	879	1.528^b^
SEM				9.48	5.82	0.011	10.5	6.51	0.011	14.1	11.0	0.010
***P*-value**												
Xy				0.897	0.517	0.671	0.940	0.781	0.778	0.530	0.682	0.088
Pb				0.057	0.038	0.949	<0.001	0.018	0.066	0.001	0.814	<0.0001
Xy × Pb				0.612	0.932	0.515	0.549	0.559	0.909	0.085	0.211	0.747

FI: feed intake, BWG: body weight gain, FCR: feed conversion ratio. FI and FCR values were standardized for 88 % dry matter.

^a-c^values within a column with no common superscripts differ significantly (*P* < 0.05).

1 CC: challenged control, Xy: challenged control+ xylanase (0.03 %), Pb: challenged control+ *B. subtilis* PB6 (0.05 %), Xy + Pb: challenged control+ xylanase (0.03 %) +*B. subtilis* PB6 (0.05 %), NC: non-challenged control.

2 NE: necrotic enteritis. ^3^SEM: standard error of mean.

**Table 5:** Effect of xylanase and *B. subtilis* PB6 on the growth performance of broilers challenged with necrotic enteritis during finisher stages (d29-35 and d20-35), and overall period (d0-35).

1Treatments	2NE Challenge	Xylanase (%)	*B. subtilis* (%)	(d29-35)			(d20-35)			(d0-35)		
				FI (g)	BWG (g)	FCR (g/g)	FI (g)	BWG (g)	FCR (g/g)	FI (g)	BWG (g)	FCR (g/g)
CC	Yes	No	No	1358	788	1.730	2791^a^	1674	1.669^a^	3958^a^	2517^b^	1.573^a^
Pb	Yes	No	Yes	1311	779	1.691	2644^b^	1649	1.606^b^	3769^b^	2475^b^	1.524^b^
Xy	Yes	Yes	No	1388	801	1.738	2755^ab^	1662	1.659^ab^	3920^ab^	2500^b^	1.568^a^
Xy+Pb	Yes	Yes	Yes	1364	803	1.704	2724^ab^	1704	1.599^b^	3841^ab^	2521^ab^	1.524^b^
NC	No			1341	790	1.706	2769^ab^	1723	1.609^b^	3951^a^	2637^a^	1.499^b^
3SEM				24.8	22.4	0.027	33.9	27.5	0.015	38.8	29.3	0.008
***P*-value**				0.279	0.933	0.764	0.032	0.324	0.004	0.005	0.004	<0.0001
**Main effect (2×2)**												
Xylanase (Xy) %												
0				1336	787	1.707	2723	1666	1.635	3868	2500	1.548
0.03				1380	806	1.717	2745	1689	1.627	3888	2517	1.544
SEM				17.3	15.3	0.020	20.0	17.6	0.011	25.2	18.9	0.011
*B. subtilis* (Pb) **%**												
0				1391^a^	802	1.727	2784^a^	1679	1.660^a^	3949^a^	2520	1.564^a^
0.05				1325^b^	791	1.696	2683^b^	1676	1.602^b^	3807^b^	2498	1.528^b^
SEM				17.3	16.3	0.021	21.2	18.6	0.011	26.6	20.0	0.011
***P*-value**												
Xy				0.080	0.378	0.726	0.420	0.360	0.538	0.597	0.529	0.603
Pb				0.010	0.642	0.349	0.003	0.894	0.001	0.001	0.457	<0.001
Xy × Pb				0.827	0.967	0.844	0.089	0.304	0.806	0.181	0.376	0.334

FI: feed intake, BWG: body weight gain, FCR: feed conversion ratio. FI and FCR values were standardized for 88 % dry matter.

^a-b^values within a column with no common superscripts differ significantly (*P* < 0.05).

1 CC: challenged control, Xy: challenged control+ xylanase (0.03 %), Pb: challenged control+ *B. subtilis* PB6 (0.05 %), Xy + Pb: challenged control+ xylanase (0.03 %) +*B. subtilis* PB6 (0.05 %), NC: non-challenged control.

2 NE: necrotic enteritis.

3 SEM: standard error of mean.

### Intestinal lesion scores

**Figure 1:**
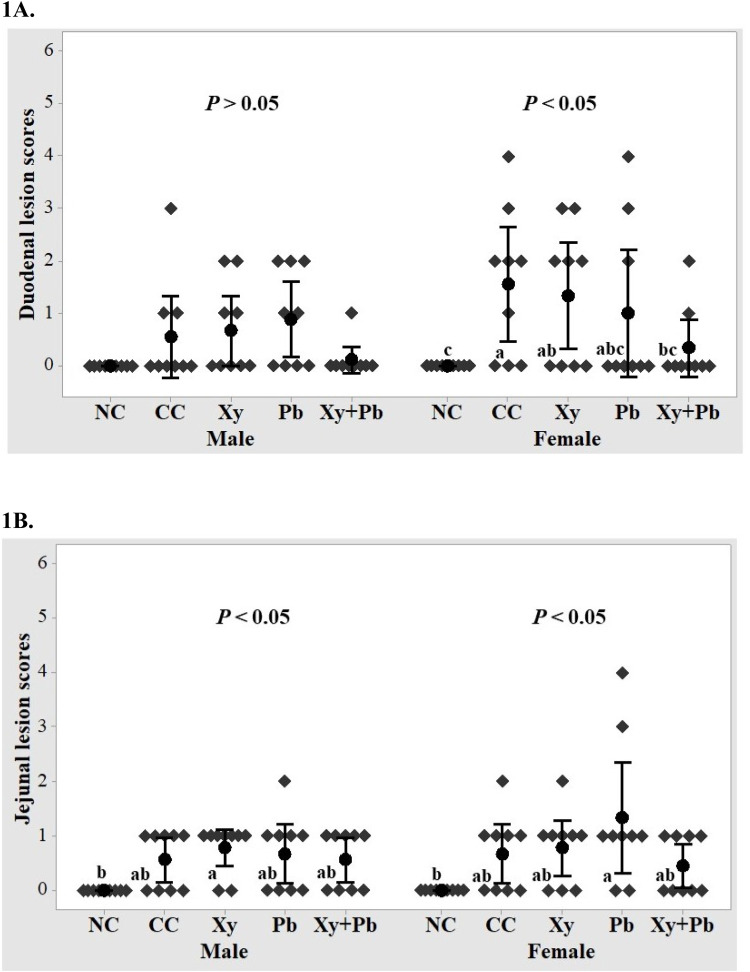
Effects of xylanase and *B. subtilis* supplementation on intestinal lesion scores (d16) of broilers challenged with necrotic enteritis. (A) male and female duodenum (B) male and female jejunum. CC: challenged control, Xy: challenged control+ xylanase (0.03 %), Pb: challenged control+ *B. subtilis* PB6 (0.05 %), Xy + Pb: challenged control+ xylanase (0.03 %) +*B. subtilis* PB6 (0.05 %), NC: non-challenged control. ^a-c^ values within a column with no common superscripts differ significantly (*P* < 0.05).

Figure 1(A-B) shows the gross lesions in 2 sections of the intestine. The NE challenge significantly increased (*P* < 0.05) duodenal lesions in female birds in the CC group compared to the NC birds. However, the Xy+Pb fed birds showed a significant reduction (*P* < 0.05) in female duodenal lesions compared to the CC birds. Additionally, the duodenal lesions of the female birds in the Pb group shifted toward the NC birds. However, jejunal lesion scores did not differ significantly (*P* > 0.05) between the CC and NC birds.

### Jejunal histomorphology

The results of histomorphometry measurements of NE-challenged birds are presented in Table 6. A significant Xy × Pb interaction (*P* < 0.05) was observed for VH and the VH/CD ratio. When administered alone, Pb significantly increased VH and improved VH/CD ratio (*P* < 0.05). However, these beneficial effects did not persist when Pb was supplemented with Xy. Moreover, a tendency of Xy × Pb interaction was observed (*P* = 0.067) with Pb showing an increased VSA when Xy was not supplemented (*P* < 0.05) but not otherwise. On the other hand, Xy supplementation as a main effect was associated with a decrease in jejunal VW (*P* < 0.01) and showed a tendency to reduce VSA (*P* = 0.054). Furthermore, all the challenged birds had increased CD (*P* < 0.001) compared to NC birds.

**Table 6:** Effects of xylanase and *B. subtilis* PB6 on jejunal villus morphology of broilers challenged with necrotic enteritis on d16.

1Treatments	2NE Challenge	Xylanase (%)	*B. subtilis* (%)	Measurements				
				Villus height (µm)	Crypt depth (µm)	Villus width (µm)	Villus surface area (mm^2^)	Villus height/Crypt depth
CC	Yes	No	No	1251^c^	338^a^	198^ab^	0.78^b^	3.73^c^
Pb	Yes	No	Yes	1536^ab^	299^a^	203^a^	0.98^a^	5.35^b^
Xy	Yes	Yes	No	1378^bc^	326^a^	177^bc^	0.77^b^	4.24^bc^
Xy+Pb	Yes	Yes	Yes	1397^bc^	333^a^	182^bc^	0.80^b^	4.28^bc^
NC	No			1664^a^	167^b^	175^c^	0.92^ab^	10.4^a^
^3^SEM				50.2	15.5	7.48	0.05	0.47
***P*-value**				<0.0001	<0.0001	0.032	0.005	<0.0001
**Main effect (2×2)**								
Xylanase (Xy) %								
0				1394	318	201^a^	0.88	4.54
0.03				1387	329	180^b^	0.78	4.26
SEM				39.4	11.7	5.34	0.03	0.24
*B. subtilis* (Pb)%								
0				1314^b^	332	188	0.77^b^	3.99^b^
0.05				1466^a^	316	192	0.89^a^	4.82^a^
SEM				39.4	11.7	5.34	0.03	0.24
***P*-value**								
Xy				0.909	0.523	0.009	0.054	0.420
Pb				0.011	0.339	0.553	0.023	0.021
Xy × Pb				0.024	0.177	0.955	0.067	0.027

^a-c^values within a column with no common superscripts differ significantly (*P* < 0.05).

1 CC: challenged control, Xy: challenged control+ xylanase (0.03 %), Pb: challenged control+ *B. subtilis* PB6 (0.05 %), Xy + Pb: challenged control+ xylanase (0.03 %) +*B. subtilis* PB6 (0.05 %), NC: non-challenged control.

2 NE: necrotic enteritis. ^3^SEM: standard error of mean.

## Discussion

Necrotic enteritis remains a significant challenge in the poultry industry, particularly in sub-clinical cases, which can hinder growth performance and feed efficiency (Hofacre et al., 2003; Hofshagen and Kaldhusdal, 1992). Traditionally, antibiotics have been the primary strategy for managing NE. However, the ban or withdrawal of in-feed antibiotics has prompted the search for alternative interventions. Therefore, the current study examined the efficacy of Xy and Pb, both individually and in combination, in broiler diets on the growth performance, liveability, intestinal lesions, and jejunal histomorphometry of birds under SNE challenge. In line with previous studies (Daneshmand et al., 2023b; Gharib-Naseri et al., 2021; Keerqin et al., 2021; Kumar et al., 2021), the successful induction of SNE was confirmed as evidenced by reduced BWG, increased FCR, increased intestinal lesions, reduced VH and VSA, VH/CD and increased CD, without significant mortality. Based on the results of the current study, we partially accepted the hypothesis that Pb alone improves performance by mitigating gut damage and promoting gut health during an SNE challenge. However, we rejected the hypothesis of Xy alone and in combination with Pb improving performance and gut health under SNE conditions.

The dietary addition of Pb significantly improved FCR in broilers under the SNE challenge, particularly during the post-recovery phase and overall period. Notably, this improvement was evident even when birds were not yet challenged (d0-8), underscoring the probiotics' potential to optimize nutrient utilization and maintain performance under SNE conditions. This improvement in feed efficiency was accompanied by significant morphological changes in the jejunum, as evidenced by increased VH, VSA, and VH/CD ratio. These changes indicate improved intestinal health and, thus, nutrient absorption, supporting the perspective that Pb supplementation plays a critical role in promoting gut function under challenging conditions. The VH/CD ratio is an important indicator of intestinal recovery and overall gut health. A higher VH/CD ratio indicates the presence of long, mature, and functional villi, along with shallow crypts, reflecting continuous epithelial cell renewal (M'Sadeq et al., 2015). This ratio serves as a reliable indicator of optimal gut morphology, with a higher ratio typically associated with improved nutrient absorption and intestinal function. These results are consistent with prior studies demonstrating that *Bacillus*-based probiotics promote nutrient absorption through improved intestinal morphology (He et al., 2019; Lee et al., 2010; Palamidi et al., 2016; Zhang et al., 2016). Additionally, the ability of Pb to reduce intestinal damage was further highlighted by its role in shifting duodenal lesion scores in NE-challenged birds toward those observed in unchallenged birds. This protective effect against NE-induced intestinal damage suggests that Pb supplementation may help mitigate the detrimental impacts of *Eimeria* and *C. perfringens* colonisation and growth in the gut. This finding aligns with previous studies by Jayaraman et al. (2013) and Ningsih et al. (2023), who reported similar protective effects of Pb against *C. perfringens*-induced intestinal damage. The potentiality of *Bacillus-*based probiotics can be attributed to their diverse mechanisms that confer probiotic benefits. These include: 1) competitive exclusion of common poultry pathogens, 2) improvement of digestion and nutrient absorption through the production of exogenous enzymes, 3) improvement of intestinal morphology, 4) modulation of the immune system, and reduction of toxic compounds (Ramlucken et al., 2020). Additionally, Pb produces bacteriocins such as subtilin, which possesses strong antibacterial properties against various pathogens including *C. perfringens* (Teo and Tan, 2005). Beyond subtilin, Pb is also known to produce other antimicrobial compounds, such as amicoumacins, (oxy) difficidin, (dihydro) bacillaene, and bacillomycin D (Jayaraman et al., 2017), further contributing to its beneficial effects. This antibacterial action can provide an insight into how this probiotic can improve intestinal health, particularly under stress conditions such as NE challenge. Interestingly, Pb’s benefits extend beyond its antibacterial activity. It has been shown that Pb can enhance gut health through mechanisms entirely different from pathogen infection (Jayaraman et al., 2013). These findings suggest that Pb could be an effective dietary probiotic for enhancing gut health, improving nutrient utilization, mitigating intestinal damage, and ultimately improving feed efficiency and recovery from SNE in broilers.

Xy supplementation did not significantly improve performance or gut health during the post-challenge phase. However, it enhanced BWG during the starter phase (d0-8), suggesting an early growth-promoting effect. During the NE challenge, *C. perfringens* produces extracellular toxins that damage the intestinal wall (Feng et al., 2022). Following the challenge, birds likely experience an increased nutrient demand for recovery and tissue repair, which Xy supplementation alone may not have been able to address fully. This is reflected in the reduced VH and VH/CD ratio and reduced VSA, indicating impaired absorptive capacity. These findings contrast with previous reports in the literature (Choct et al., 2004; Gao et al., 2008; Hosseini et al., 2017), that reported positive growth responses to Xy supplementation in a wheat-based diet following the NE challenge. One possible explanation is diet composition, as Xy is typically known to be more effective in wheat-based diets due to their higher NSP content (Kiarie et al., 2014). Furthermore, the quality of the corn used in this study may also have played a role in the lack of significant effects, as lower-quality (high-fiber) corn has been shown to respond better to Xy supplementation (Petry et al., 2019). The combination of Pb and Xy improved FCR during the starter, finisher, and overall periods and reduced duodenal lesions in female birds. However, these effects did not exceed those observed with Pb supplementation alone, indicating the absence of synergistic effects between Xy and Pb under SNE challenge conditions. This finding aligns with the results of Machado et al. (2020), who also reported no associative effect of xylanase and probiotics on broiler performance. In contrast, Wang et al. (2021) observed increased VH and a higher VH/CD ratio when combining enzymes (xylanase and β-glucanases) with *B. subtilis* DSM 29784. The discrepancy might be due to different *Bacillus* strains and multiple enzyme combinations used. Xy and Pb target gut health through distinct mechanisms: Xy acts on feed NSP, indirectly benefiting the gut microbiome (Vasanthakumari et al., 2023), while Pb directly influences the gut microbiome (Memon et al., 2022; Teo and Tan, 2006) to maintain a healthy gut. Their combined use contributes to the proliferation of beneficial bacteria and suppresses the pathogens. However, their combined effects may not surpass those of the individual additives due to overlapping mechanisms, limiting the synergetic effect. This information can be important for Xy dose selection when combined with Pb.

The starter phase is a critical period in the broiler production cycle, laying the foundation for growth and immune function throughout the production cycle. In this study, during the starter phase (d0-8), birds were not yet challenged. The dietary addition of Pb alone significantly reduced FCR in this phase. However, previous studies using a similar Pb strain with different doses in corn-based diets have reported contrasting results. For instance, no significant improvement in FCR was observed during d0-10 (Ciurescu et al., 2020), d0-21 (Aljumaah et al., 2020), d0-16 (Abudabos, 2013), and d1-18 (Liu et al., 2021a). In this study, FCR improvement during the starter phase, potentially due to the enzymatic activity of *B. subtilis*, which produces amylase (Ibrahim et al., 2012), protease (Olajuyigbe and Ajele, 2005), and lipase (Shah, 2011), enhancing nutrient digestion and absorption. The difference between the results of this study and previous studies may be attributed to differences in the dosing of Pb and variations in rearing conditions. These findings suggest that Pb supplementation with the current dose can support improved feed efficiency during the early stages of broiler production, making it a valuable strategy for optimizing feed efficiency during this critical period.

In this study, BWG significantly increased during the starter phase (d0-8) when birds were fed Xy alone without affecting FI. Previous studies demonstrated the positive and negative effects of Xy during the early stages of broiler growth. Consistent with our findings, Zhang et al. (2014) and Chiang et al. (2005) reported improved BWG with Xy supplementation from d0-21 in wheat-based diets and diets where 25 % of corn was replaced with wheat, respectively. However, contrasting results were observed by Daneshmand et al. (2023a) and Stefanello et al. (2017), who found no significant effects of Xy on BWG during d0-8 and d0-7 with wheat and corn-based diets, respectively. The differences in these findings may be related to the varying dietary compositions, as the total NSP content of wheat is approximately 11 %, while that of corn is 9 % (Knudsen, 2014). Moreover, soluble NSP comprises 19 % of the total in wheat (Rodehutscord et al., 2016) but only less than 2 % in corn (Choct, 1997; Kiarie et al., 2014). It is well accepted that diets with higher soluble NSP content, such as wheat, are generally more likely to benefit from Xy supplementation. However, young birds are particularly sensitive to both soluble and insoluble NSP (Kiarie et al., 2014). Therefore, they are likely to benefit from supplemental Xy regardless of the cereal type in the diet, as the benefit is likely due to hydrolysis of arabinoxylans (AXs), key cell wall components. The breakdown of these AXs alters the cell wall structure and releases encapsulated nutrients, making them more accessible for digestion and absorption. The ability of Xy to hydrolyze corn-based AXs into soluble, more fermentable fractions has been confirmed in pigs (Kiarie et al., 2016) and in an in vitro study (Pedersen et al., 2015). This mechanism could underlie the improved BWG through increased nutrient availability observed in corn-based diets in this study. Xy supplementation likely increases nutrient availability despite corn having a lower soluble NSP content than wheat. However, the lack of significant improvements in FCR during the starter phase may suggest that the benefits of Xy supplementation are more pronounced in terms of growth rather than feed utilization efficiency.

In conclusion, this study elucidated the effects of Xy and Pb on broilers under SNE challenge conditions. Supplementation with Pb and Xy alone led to a significant positive impact by improving FCR and BWG, respectively, in the pre-challenge state during the starter phase. Importantly, Pb alone also improved FCR during the post-challenge and overall period, highlighting its potential as a beneficial probiotic for supporting gut health in poultry diets. Furthermore, the combination of Xy and Pb did not yield additional benefits, suggesting overlapping mechanisms that may limit synergistic effects. Consequently, further research is warranted to investigate the effects of varying Xy dosages, both alone and in combination with Pb, to optimize their impact on broiler performance and gut health under NE challenge conditions. The findings of this study offer practical insights into the use of Pb and Xy as potential antibiotic alternatives to in-feed antibiotics for managing necrotic enteritis in broiler production. They could support the development of antibiotic-free feeding programs aimed to improve gut health, boost resistance to enteric pathogens, and ultimately enhance flock performance in commercial poultry operations.

## Disclosures

The authors declare that they have no known competing financial interests or personal relationships that could have appeared to influence the work reported in this paper.

The authors declare the following financial interests/personal relationships which may be considered as potential competing interests:

Shu-Biao Wu reports financial support was provided by Kemin Animal Nutrition and Health, Asia Pacific. If there are other authors, they declare that they have no known competing financial interests or personal relationships that could have appeared to influence the work reported in this paper.

## Appendix Supplementary materials

Image, application 1
