# Supplementary material for: Potential of Bacillus subtilis PB6 in corn-based diets to combat subclinical necrotic enteritis in broilers
Source: Poult Sci. 2025 Jul 13;104(10):105574. doi: 10.1016/j.psj.2025.105574 (PMC12341601; doi:10.1016/j.psj.2025.105574)
Supplement: Supplementary file 1 [file mmc1.docx]

| **^1^Treatments** | **Liveability, %** | | | |
| --- | --- | --- | --- | --- |
|  | **d0-8** | **d9-19** | **d20-35** | **d0-35** |
| CC | 99.2 | 100 | 96.3 | 96.1 |
| Pb | 100 | 100 | 96.1 | 96.7 |
| Xy | 100 | 97.6 | 98.6 | 96.6 |
| Xy+Pb | 100 | 99.2 | 94.7 | 94.9 |
| NC | 100 | 98.5 | 96.8 | 95.8 |
| ^2^SEM | 0.29 | 0.82 | 1.78 | 1.78 |
| *P*-value | 0.501 | 0.159 | 0.702 | 0.958 |

**Table 7**: Effect of xylanase and *Bacillus subtilis* on the liveability of birds under necrotic enteritis challenge

^1^CC: challenged control, Xy: challenged control+ xylanase (0.03%), Pb: challenged control+ *B*. *subtilis* PB6 (0.05%), Xy + Pb: challenged control+ xylanase (0.03%) +*B*. *subtilis* PB6 (0.05%), NC: non-challenged control. ^2^NE: necrotic enteritis. ^2^SEM: standard error of mean. ^a-c^ values within a column with no common superscripts differ significantly (*P* < 0.05).
